# High-Temperature Isostructural Phase Transition in Ce_2_(MoO_4_)_3_: A Rare Phenomenon Investigated through X‑ray Diffraction and Raman Scattering

**DOI:** 10.1021/acsomega.5c03153

**Published:** 2026-01-06

**Authors:** Zeyna dos Santos Viegas, Alan Silva de Menezes, Cleânio Luz-Lima, Paulo de Tarso Cavalcante Freire, Clenilton Costa Santos, João Victor Barbosa Moura

**Affiliations:** † Departamento de Física, Centro de Ciências Exatas e Tecnologia, Universidade Federal do Maranhão, CEP 65080-800 São Luís, MA, Brazil; ‡ Departamento de Física, Campus Ministro Petrônio Portella, Universidade Federal do Piauí, CEP 64049-550 Teresina, PI, Brazil; § Departamento de Física, Campus do Pici, Universidade Federal do Ceará, CEP 60455-760 Fortaleza, CE, Brazil

## Abstract

The rare-earth compound cerium­(III) molybdate (Ce_2_(MoO_4_)_3_) has gained attention due to its diverse industrial applications, such as photocatalysis, corrosion inhibition, self-repair of protective layers, as well as antiviral and antibacterial properties. However, its response to extreme temperature conditions remains insufficiently explored. This study employs ambient powder X-ray diffraction (PXRD), UV–vis diffuse reflectance spectroscopy, scanning electron microscopy (SEM), and Raman spectroscopy techniques to confirm the successful hydrothermal synthesis of a crystalline sample of the Ce_2_(MoO_4_)_3_ compound. Subsequently, in situ temperature-dependent (13–973 K) XRD and Raman scattering (293–998 K) studies were conducted. Rietveld analysis of diffraction patterns reveals a stable low-temperature phase (13–303 K) and anomalies in the high-temperature evolution (*T* > 583 K) of lattice parameters and the sample’s microstrain and crystallite size. In the high-temperature (*T* > 848 K) Raman spectra of Ce_2_(MoO_4_)_3_, an additional band emerges at 452 cm^–1^; the observed anomalies are attributed to an isostructural phase transition (IPT). This type of phase transition is among the rarest phenomena reported in the literature. Our findings enhance the understanding of the physical properties of Scheelite-type compounds and emphasize the importance of investigating these uncommon phenomena. This knowledge can potentially drive the development of novel materials and expand their applications in materials science.

## Graphical abstract

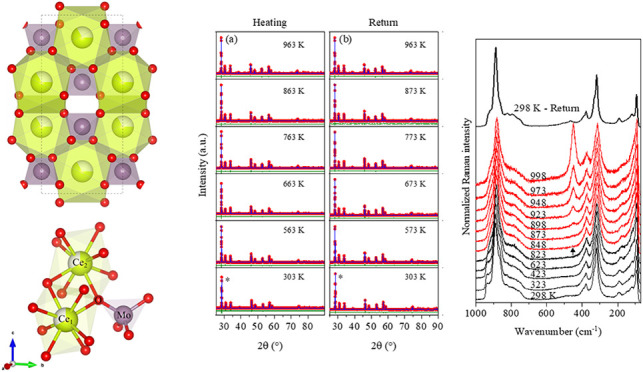

## 1 Introduction

The study of phase transitions in crystalline materials has been a central theme in condensed matter physics and materials science due to its profound implications for the understanding of material properties and tailoring of functional applications. In 1937, Landau provided a phenomenological approach for describing phase transitions based on symmetry changes as a necessary condition for the appearance of a new phase.^1^ Within this framework, first-order transitions are marked by discontinuities in observable quantities, while second-order transitions evolve continuously as the order parameter changes smoothly. However, in 1949, Lawson et al. observed a 16.5% volume collapse in metallic cerium under high pressure,^2^ accompanied by 4f → 5d electron delocalization and a sharp decrease in magnetic susceptibility, without changes in crystal symmetry. This discovery revealed the existence of phase transitions outside Landau’s classification. In 1980, Cowley expanded the theoretical framework to include such symmetry-preserving transitions, introducing Type 0 (Cowley-type) phase transitions, now known as isostructural phase transitions (IPTs).^3^ Later, Christy et al. 1995 showed that IPTs correspond to multiple local minima of Landau’s order parameter, which may cause the equilibrium line to extrapolate into a crossover line,^4^ allowing phase coexistence. As such, material properties vary rapidly, and limited instrumental resolution may render the discontinuity experimentally indistinct.

Several experimental techniques can be used to detect IPTs. The aforementioned metallic cerium IPT was detected by X-ray diffraction (XRD) studies, where the lattice parameters exhibited 16.5% volume collapse under high pressure;^2^ the semiconductor SmS IPT was detected by thermoelectric measurements, where anomalies are observed in the thermos power vs pressure graph along selected isothermal curves;^5^ the PbCrO_3_ perovskite exhibits significant volume collapse without symmetry changes under high pressure (≈3 GPa), which leads to an insulator–metal Mott transition, as confirmed by dielectric measurements;^6^ monoclinic wolframite-type InNbO_4_ exhibits large volume collapse in high pressure (≈10.8 GPa), detected by synchrotron-based XRD and Raman spectroscopy studies.^7^ From these studies, IPTs can be detected by observing anomalies in the established trends (either expected or initially observed) in the parameters’ evolution with extensive thermodynamic parameters.

Among these materials, Scheelite-type rare-earth molybdates (RE_2_(MoO_4_)_3_) are promising candidates for the study of IPTs. The propensity of these structures to undergo phase transitions is well-documented in related families, such as the pressure-induced scheelite-to-fergusonite transition observed in scheelite-type perrhenates.^8^ The lanthanide rare-earth series, in particular, belongs to the defect scheelite-type family, of composition Ln_0.667_[MoO_4_] (Ln = Ce, Pr, Nd, Sm). They crystallize in the *I*4_1_/*a* tetragonal space group, with the Ln^3+^ cation and Mo^6+^ centers occupying the 4b and 4a Wyckoff positions, respectively. Due to them sharing the same site symmetry (4), charge neutrality is ensured with partial occupancy (∼0.667) of the Ln^3+^ atoms.^9^ The unique electronic configuration of cerium, which alternates between Ce^3+^ and Ce^4+^ states^10^ in extreme thermodynamic conditions,^11^ singles out cerium-based defect scheelite-type materials as potential candidates for occurrence of IPTs, as elemental cerium was the first reported instance of an IPT.

Investigations into potential phase transitions in scheelite-type cerium-based molybdates have been carried out by Saha et al. and Moura et al. on the LiCe­(MoO_4_)_2_ (LCM) and NaCe­(MoO_4_)_2_ (NCM) materials, respectively,^12,13^ employing powder X-ray diffraction (PXRD) and Raman scattering techniques. Both studies have identified IPTs occurring in high-temperature regimes with distinct mechanisms proposed for each transition. Saha et al. argues that the transition in the LCM material is driven by the nucleation and growth of a new phase within the matrix of the room-temperature phase. In contrast, Moura et al. proposed that in the NCM material the mechanism driving the phase transition involves the random redistribution of sodium and cerium atoms within the unit cell. Additionally, Ramarao et al. reported an IPT on the scheelite-type CaMoO_4_ based on anomalies identified in differential scanning calorimetry and dielectric studies in high-temperature regimes.^14^

Despite notable progress, the high-temperature behavior of defect scheelite-type structures such as Ln_0.667_[MoO_4_] remains insufficiently explored when compared to that of high-pressure studies. In this work, we investigate the cerium molybdate Ce_2_(MoO_4_)_3_ (CMO) scheelite phase through an integrated approach combining PXRD, Raman spectroscopy, scanning electron microscopy (SEM), and diffuse reflectance measurements. We begin with a comprehensive room-temperature characterization of its structural, vibrational, morphological, and electronic properties, followed by in situ temperature-dependent PXRD and Raman scattering analyses as well as ex situ diffuse reflectance studies, to probe potential phase transitions. This work advances the fundamental understanding of isostructural phase transitions in scheelite-type molybdates and highlights the potential of these materials for future technological applications in catalysis, optoelectronics, and energy storage.

## 2 Experimental Procedures

### 2.1 Synthesis

The Ce_2_(MoO_4_)_3_ sample was prepared with the hydrothermal method, using a stoichiometric ratio of 3:2 of ammonium molybdate tetrahydrate ((NH_4_)_6_Mo_7_O_24_·4H_2_O, 99.98% purity, Sigma-Aldrich) and cerium­(III) nitrate hexahydrate, (Ce­(NO_3_)_3_·6H_2_O, 99% purity, Sigma-Aldrich). Both reactants were separately dissolved in 50 mL of distilled water. The solutions were stirred for 30 min, after which the Ce^3+^ solution was slowly added to the Mo^6+^ solution. The pH level was adjusted to 7 using a 5 mol/L solution of sodium hydroxide, and the resulting solution was stirred for an additional 30 min. The solution was then transferred to a 100 mL Teflon-lined stainless steel autoclave reactor and heated at 160 °C for 6 h. Following the hydrothermal treatment, the solution was allowed to cool naturally to room temperature. The resulting product was washed several times with distilled water and ethanol. Finally, the yellow powder was collected and dried in an air atmosphere at 90 °C for 24 h.

### 2.2 Characterization

Structural characterization was carried out with the PXRD technique, using a Bruker’s D8 Discover diffractometer, equipped with the LynEye XE linear detector, operating at 40 kV/40 mA and using Cu Kα_1_ radiation of λ_1_ = 1.5406 and Cu Kα_2_ radiation of λ_2_ = 1.5440 Å. Measurements were taken in the 2θ range from 25 to 90° with a step size of 0.02°/min, under an air atmosphere. The lattice parameters were obtained using the Rietveld refinement^15^ with the General Structure Analysis System (GSAS II) software.^16^ The background was fitted by using a shifted Chebyshev polynomial of the first kind (10 coefficients). Ce occupancy was fixed at 0.667, while the Mo and O sites were fixed to full occupation (1.0). Refinement proceeded sequentially for lattice parameters, scale factor, sample displacement, oxygen fractional coordinates, preferred orientation (modeled with a generalized spherical harmonics of second order), isotropic displacement parameters (*U*_iso_), crystallite size (Lorentzian broadening), and microstrain (Gaussian broadening). The pseudo-Voigt function was used to model the peak shape.

The optical properties of the sample were investigated under an air atmosphere using the UV–vis diffuse reflectance spectroscopy technique. The reflectance spectrum was acquired with a UV-3600 Shimadzu spectrophotometer equipped with an integrating sphere (model ISR-3100) accessory operating in the diffuse reflection mode. A magnesium sulfate (MgSO_4_) standard was used as a white reference. Vibrational analysis was conducted with a Raman spectroscopy technique. The Raman spectrum of the sample was measured using a T64000 Horiba spectrometer, equipped with a solid state λ = 532 and 633 nm laser. The beam was focused on the sample surface using an Olympus BX41 microscope equipped with a 20× long working distance (20.5 mm) objective lens with a numerical aperture of 0.35. The slit width was set to a resolution of 2 cm^–1^. The spectrum was collected with five accumulations and an acquisition time of 60 s over the 1100–70 cm^–1^ range, under air atmosphere. The surface morphology of the CMO material was examined using a Zeiss EVO HD15 high-resolution scanning electron microscope under an air atmosphere.

Temperature-dependent structural characterization was conducted across both low- and high-temperature regimes with the PXRD technique. At low temperatures, the diffraction patterns were obtained in the 300–13 K range, under vacuum, using an Oxford Cryosystems PheniX temperature chamber. At high temperatures, patterns were obtained in the 300–973 K range using an Anton-Paar HTK 1200N temperature chamber. The high-temperature measurements were conducted under an ambient air atmosphere. Temperature-dependent Raman scattering measurements were conducted in the high-temperature regime using a Linkam thermal stage THS 1200, spanning the range from 300 to 998 K, under an air atmosphere. Spectral data were acquired after each temperature increment, following a stabilization time of 15 min.

## 3 Results and Discussion

### 3.1 Room-Temperature PXRD Analysis

Figure 1 presents the observed PXRD pattern (red dots) of the synthesized CMO sample. The diffraction pattern reveals strong, sharp, and well-defined peaks with low background intensity, characteristic of crystalline materials. All reflections were indexed (green bars) to the scheelite-type tetragonal structure in the *I*4_1_/*a* space group, corresponding to the Inorganic Crystal Structure Database (ICSD) card No. 423509.^9^ The absence of additional reflections confirms the successful synthesis of the sample, with no evidence of secondary phases or impurities.

**Figure 1:**
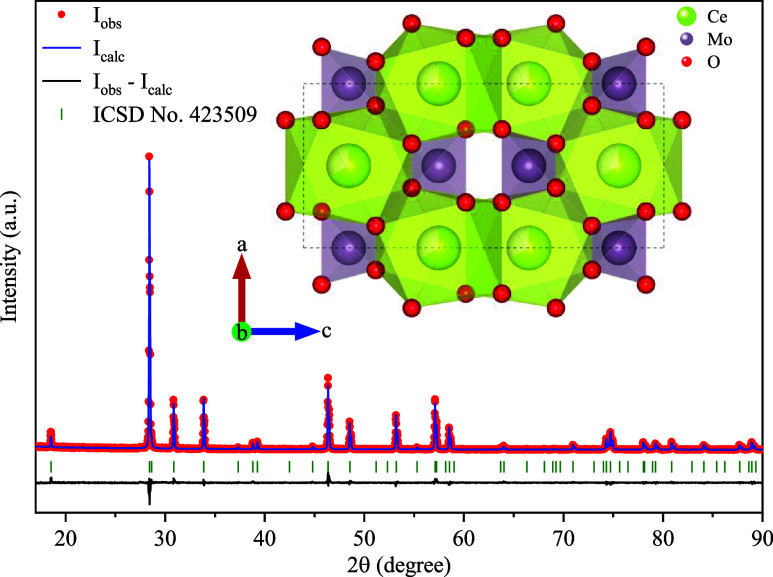
Powder X-ray diffraction pattern of the Ce_2_(MoO_4_)_3_ powder obtained by the hydrothermal method. An inset representation of the crystal structure of the CMO crystal at room temperature shows the [MoO_4_] and [CeO_8_] groups.

The lattice parameters and unit cell volume of the crystalline system were obtained with the Rietveld refinement method.^17^ The Rietveld refinement confirms the defect scheelite-type nature of the structure, with cerium atoms partially occupying the 4b Wyckoff site with an occupancy of 0.667(6), consistent with the CMO stoichiometry. This partial occupancy is a distinctive feature of the defect scheelite-type family Ln_0.667_[MoO_4_], where the under-occupation of the Ln^3+^ site locally perturbs the ideal symmetry, potentially lowering the effective site symmetry and influencing the vibrational properties of the crystal. Table 1 summarizes the lattice parameters, atomic coordinates, and corresponding *R*-values for the refinement.

**Table 1:** Lattice Parameters, Quality Indicators of Structural Refinement (*R*-Values), Site Occupation and Atomic Coordinates Obtained by the Rietveld Refinement of Ce_2_(MoO_4_)_3_ Crystals

lattice parameters	*R*-values	atoms	occupancy	Wyckoff	*x*	*y*	*z*
*a* = *b* = 5.3201(3) Å	*R*_wp_ = 11.95%	Ce	0.667	4b	0	0.25	0.625
*c* = 11.6590(9) Å	GOF = 1.204	Mo	1	4a	0	0.25	0.125
Vol = 329.992(3) Å^3^	χ^2^ = 1.445	O	1	16f	0.1379(9)	0.0048(8)	0.2031(3)

The inset of Figure 1 includes a representation of the CMO unit cell, modeled with the VESTA software,^18^ using the lattice parameters and atomic positions obtained in the refinement. In this structure, the Ce and Mo atoms are both on fixed special positions of site symmetry at (0, 1/4, 5/8) and (0, 1/4, 1/8), respectively. This is arrangement is analogous to the positions occupied by the Ca and W atoms in the CaWO_4_ scheelite;^19^ partial occupancy of the Ce atom at the 4b site classifies the Ce_2_(MoO_4_)_3_ material as a defect scheelite-type, as described by Schustereit et al.^9^ As such, it can be visualized as the assembly of Ce dodecahedral and Mo tetrahedral units.

Figure 2 shows the isolated dodecahedral and tetrahedral units that compose the CMO unit cell. Ce atoms are coordinated by eight oxygen atoms, forming distorted trigonal dodecahedral [CeO_8_] sites, while molybdenum atoms are coordinated by four oxygen atoms, forming bisphenoidally distorted tetrahedral [MoO_4_] sites. The refined lattice parameters and coordination environments are consistent with those observed in previously reported scheelite-type materials;^9,19–21^ subtle differences, such as smaller unit cell volume, may be attributed to different synthesis route and conditions.

**Figure 2:**
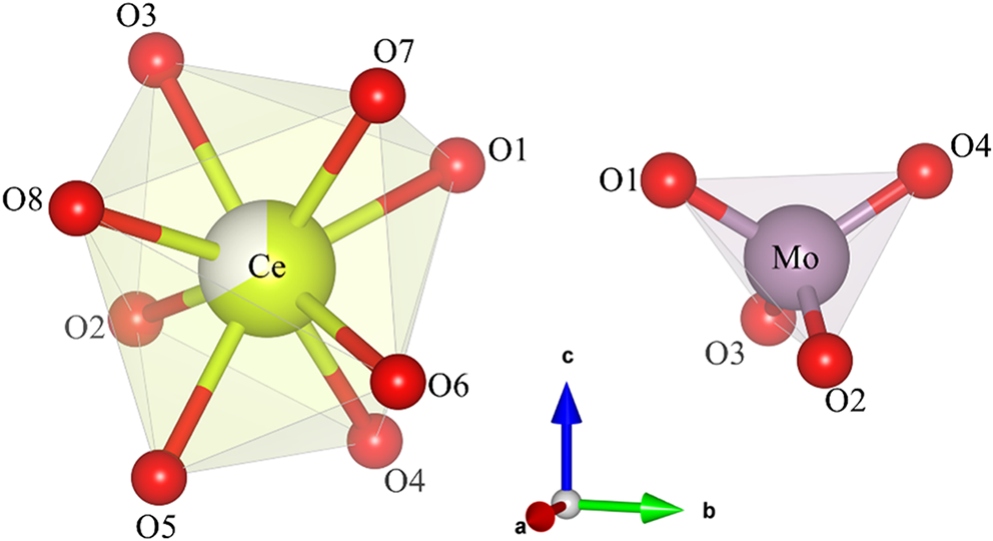
Isolated trigonal dodecahedral [CeO_8_] and bisphenoidal tetrahedral [MoO_4_] groups present in the Ce_2_(MoO_4_)_3_ crystals.

Ce–O and Mo–O bond lengths are listed in Table 2 as well as the O–Mo–O bond angles (atom numbering corresponding to Figure 2). The [MoO_4_] exhibit equal Mo–O bond lengths (4 × 1.757(2) Å), consistent with tetrahedral coordination; however, their internal O–Mo–O bond angles deviate from the ideal (6 × 109.5°) tetrahedral internal angles, splitting into four narrower angles (105.3(2)) and two wider angles (118.1(2)). This distortion is further seen in the [CeO_8_] dodecahedra, with four shorter Ce–O bond lengths (2.528(3)) and four longer bond lengths (2.539(4)) as well as eight distinct internal O–Ce–O angles (Table S1). The observed distortion is topologically mandated by the snub-disphenoid geometry of the trigonal dodecahedron,^9^ which can be exacerbated by defects in the structure.^22^

**Table 2:** Internal Cerium–Oxygen and Molybdenum–Oxygen Bond Lengths, as well as Molybdenum–Oxygen Bond Angles of the Coordination Environment of Ce_2_(MoO_4_)_3_ Crystals^a^

Ce–O	bond lengths (Å)	*d*(O_1_–Ce) = *d*(O_2_–Ce) = *d*(O_6_–Ce) = *d*(O_8_–Ce)	2.528(3)
		*d*(O_3_–Ce) = *d*(O_4_–Ce) = *d*(O_5_–Ce) = *d*(O_7_–Ce)	2.539(4)
Mo–O	bond lengths (Å)	*d*(O_1_–Mo) = *d*(O_2_–Mo) = *d*(O_3_–Mo) = *d*(O_4_–Mo)	1.757(2)
	bonds angles (deg)	∠(O_1_–Mo–O_2_) = ∠(O_1_–Mo–O_3_) = ∠(O_2_–Mo–O_4_) = ∠(O_3_–Mo–O_4_)	105.3(2)
		∠(O_1_–Mo–O_4_) = ∠(O_2_–Mo–O_3_)	118.1(2)

a The *d* symbol represents distance and ∠ represents angle.

Table 3 displays comparisons between the bond lengths and angles of the CMO system with those of ideal CaWO_4_ scheelite (ICSD No. 18135 ^23^). Table S2 shows the relative deviations, which were calculated using Δ = (1 – *P*_CWO_/*P*_CMO_) × 100, where *P* represents bond lengths (*d*) or angles (∠) of either system. Due to the Ce and Mo atoms occupying sites with equal multiplicity (4a and 4b, respectively), the structure achieves electrical neutrality with the under-occupation of the 4b site.^9^ Therefore, the oxygen atoms are pulled toward the neighboring Mo atoms, which explains the shorter Mo–O (Δ ≈ −0.797%) and longer Ce–O_1,3_ (Δ_1,3_ ≈ 2.324 and 3.129%, respectively) bond lengths observed in Table 3. Consequently, there is a slight flattening of the tetrahedral units, as the angular deviations show a narrowing in the smaller angle (Δ ≈ −1.8%) and a concomitant widening of the longer angle (Δ ≈ 3.4%).

**Table 3:** Relative Deviation of Ce_2_(MoO_4_)_3_ Material’s Bond Lengths and Internal Bond Angles from CaWO_4_ Scheelite^23^ ^,^ ^a^

	A = Ce	A = Ca^23^	
parameters	B = Mo	B = W^23^	Δ (%)
*d*(O_1_–A) (Å)	2.528(3)	2.449(11)	3.129
*d*(O_3_–A) (Å)	2.539(4)	2.480(11)	2.324
*d*(O_1_–B) (Å)	1.7589(11)	1.771(11)	–0.797
∠(O_1_–B –O_2_) (deg)	105.4(2)	107.2(4)	–1.7
∠(O_1_–B–O_4_) (deg)	118.0(2)	114.1(7)	3.3

a The notation *d*(O_1_–A) stands for distance between O_1_ and atom A (site 4b) or B (site 4a), while the notation ∠ stands for angle.

The dodecahedra’s internal angles display pronounced internal angle distortions (Table S2), with considerable narrowing of the ∠(O_2_–Ce–O_4_) and ∠(O_3_–Ce–O_7_) angles (Δ ≈ −3.3%), located on opposite sides of the polyhedron (see Figure 2). This concurrently widens the ∠(O_2_–Ce–O_6_) and ∠(O_3_–Ce–O_4_) angles (Δ ≈ 2.7%), so the overall structure maintains its effective coordination number of 8.

Next, we examine the interpolyhedral Ce–O–Mo bond angles. Each oxygen atom is bonded to one Mo atom and two Ce atoms, as shown in Figure 3. In the ideal CaWO_4_ structure, the Ca_1,2_–W–O bond angles are 120.5(5) and 132.1(6)°, respectively, while in the defect scheelite-type Ce_0.667_MoO_4_ obtained, the angles from the same crystallographic unique atomic positions are 122.1(2) and 133.6(2)°. The relative deviations are Δ_1_ ≈ 1.1 and Δ_2_ ≈ 1.5%, showing slight widening. We conclude that the overall impact of the partial occupation of the 4b site by the Ce^3+^ cation results in a 2-fold distortion of the system’s polyhedral units: the shortening of the Mo–O bonds (Δ ≈ −0.797) and slight flattening of the [MoO_4_] tetrahedra (Δ ≈ −1.8 and 3.4%), followed by the elongation of Ce–O bonds (Δ_1,3_ ≈ 2.324 and 3.129%) and severe angular distortions on the [CeO_8_] dodecahedra.

**Figure 3:**
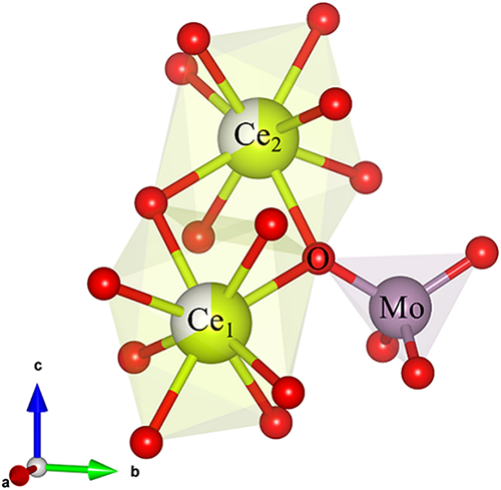
Interpolyhedral connections in the Ce_2_(MoO_4_)_3_ material. Each oxygen atom is uniquely bonded to two Ce atoms and one Mo atom.

### 3.2 Room-Temperature Raman Scattering Analysis

Scheelite-type compounds with the general formula ABO_4_ (A = Ba, Ca, Cd, Sr, Pb; B = Mo, W) crystallize in the *I*4_1_/*a* space group, exhibiting *C*_4*h*_ point-group symmetry and containing two formula units per primitive cell. For the *N* = 12 atoms present in the primitive cell, there are 3*N* = 36 vibrational degrees of freedom. However, in the defect scheelite-type CMO phase, the partial occupancy (0.667(6)) of Ce^3+^ ions at the 4b sites reduces the effective number of atoms per primitive cell to *N* = 11.3(3), corresponding to 3*N* = 34 degrees of freedom. Group theoretical analysis predicts a total of 13 zone-center Raman-active modes:

1 $$\Gamma =3A_{g}+5B_{g}+5E_{g}$$

Assignment of the modes observed in the CMO spectrum was carried out by comparison with the assignments performed in the literature for similarly structured materials.^12–14,20,24^ Figure 4 shows the experimental (red circles) Raman spectrum of the CMO powder obtained at room temperature in the 1000–70 cm^–1^ spectral range, where 15 bands are observed. The fitting procedure was performed using the Fityk software,^17^ where the peaks were modeled using a convolution of Lorentzian and Gaussian functions (the Voigt profile provides the best fit for the symmetric Raman bands of crystallite materials^25^), employing least-squares refinement.

**Figure 4:**
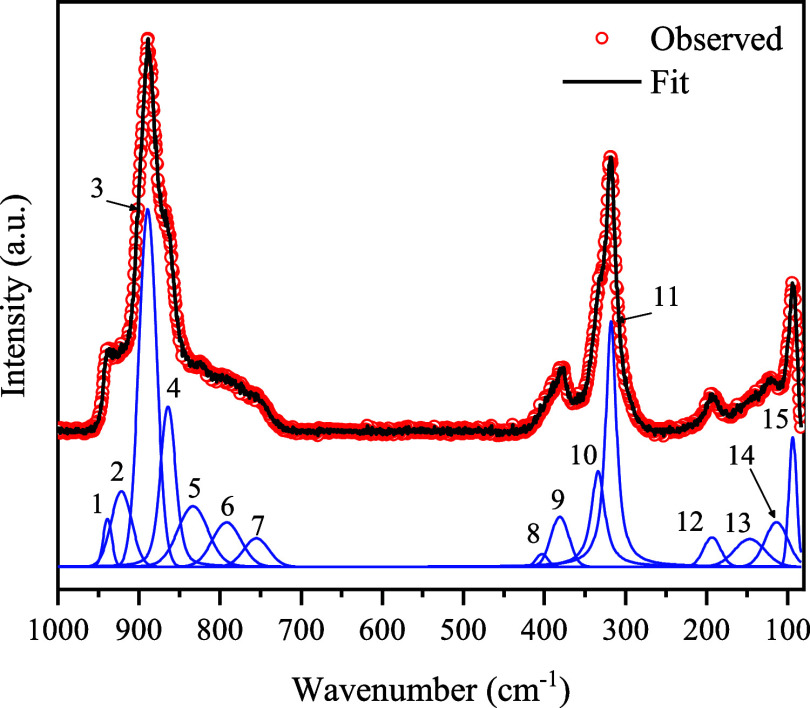
Raman spectrum of the Ce_2_(MoO_4_)_3_ powder at room temperature and fitted Voigt functions.

Three distinct band regions can be seen in the room-temperature spectrum of the CMO material. The peaks in the 1000–600 cm^–1^ range are ascribed to symmetric and antisymmetric stretching vibrations (ν_1_ and ν_3_) of the [MoO_4_]^2–^ tetrahedra; peaks in the 600–300 cm^–1^ range correspond to the symmetric and antisymmetric bending modes (ν_2_ and ν_4_) of the O–Mo–O bonds, and peaks below 300 cm^–1^ are external modes, associated with translations and librations of both the tetrahedral and Ce^3+^ units.^13,20,24,26,27^

Out of the 15 observed bands, 13 correspond directly to the vibrational modes (Table 4) reported in other scheelite-type materials.^12–14,20^ The additional two bands at 755 cm^–1^ (peak no. 7) and 864 cm^–1^ (peak no. 4) could not be reliably assigned to any previously reported symmetric and antisymmetric stretching vibrational modes. The appearance of these bands may be caused by local lowering of symmetry induced by under-occupation of the 4b site by the Ce atoms. As such, (i) the degeneracy of the E_g_ mode can be lifted, splitting into two bands, (ii) B_u_ silent modes can be activated due to distortions on the [MoO_4_]^2–^ tetrahedra, or (iii) multiple distinct local configurations of the MoO_4_ units may arise, each slightly altering the frequencies of the ν_1_ and ν_3_ modes, thereby producing an envelope of components in the Raman spectrum.

**Table 4:** Raman Modes of the Ce_2_(MoO_4_)_3_ Crystals Observed Wavenumbers and Their Assignments^a^

peak no.	ω_obs_ (cm^–1^)	modes assignment
1	939	combination^24^
2	922	combination^24^
3	889	ν_1_(A_g_)^12,13,20^
4	864	
5	833	ν_3_(B_g_)^20^
6	792	ν_3_(E_g_)^20^
7	755	
8	403	ν_2_(E_g_)^14^
9	381	ν_4_(B_g_)^20^
10	334	ν_2_(B_g_)^14^
11	318	ν_2_(A_g_)^20,24^
12	193	R([MoO_4_]^2–^)^24^
13	147	lattice modes^24^
14	114	lattice modes^24^
15	94	lattice modes^24^

a ν_1_, ν_3_, ν_2_, and ν_4_ denote symmetric stretching, asymmetric stretching, symmetric bending and asymmetric bending vibrations of MoO_4_ units, respectively. R denotes rotational modes of [MoO_4_]^2+^ tetrahedra. Lattice modes refer to translations and librations of both the tetrahedral and Ce^3+^ units.

### 3.3 Morphology Analysis

SEM images of the CMO sample are presented in Figure 5a,b. The micrographs reveal a morphology of irregular elongated plates forming flower-like aggregates. In the low-magnification image of Figure 5a, these aggregates appear as compact clusters of overlapping plates, while in the high-magnification image of Figure 5b, the individual plates become more apparent. The absence of different morphology aggregates indicates that the synthesis procedure yielded a sample of exceptional purity and quality.

**Figure 5:**
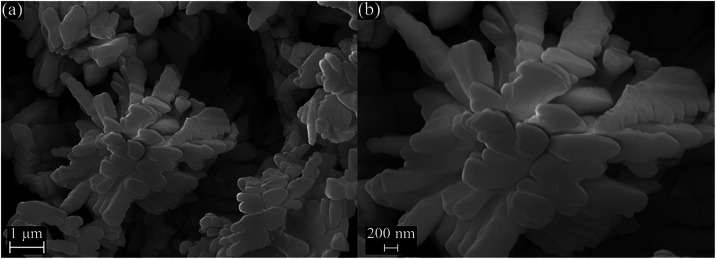
(a) SEM image of the Ce_2_(MoO_4_)_3_ sample synthesized by the hydrothermal method and (b) the magnified view on an aggregate, revealing finer surface detail.

### 3.4 UV–Vis Diffuse Reflectance Spectroscopy Analysis

The diffuse reflectance spectra of the CMO sample before (RT) and after (HT) the heating cycle (*T*_max._ = 973 K) are shown in the insets of Figure 6. At wavelengths below ∼400 nm, the incident photons have enough energy to generate electron–hole pairs, resulting in strong UV absorption by the RT and HT materials, evidenced by the flat (*R* ≈ 5%) portion of the reflectance curve. At wavelengths above ∼600 nm, the energy of the incident photons no longer promote pairs, resulting in low absorption by the RT and HT materials, evidenced by the flat (*R* ≈ 70 and ≈ 60%, respectively) portion of the reflectance plot. The strong absorption in the UV–blue regions (200–500 nm) and high reflectance in the yellow-red regions (500–700 nm) imparts a yellowish coloration to the powder, consistent with Ce^3+^-based molybdates,^20,28–33^ remaining unchanged after the heating cycle.

**Figure 6:**
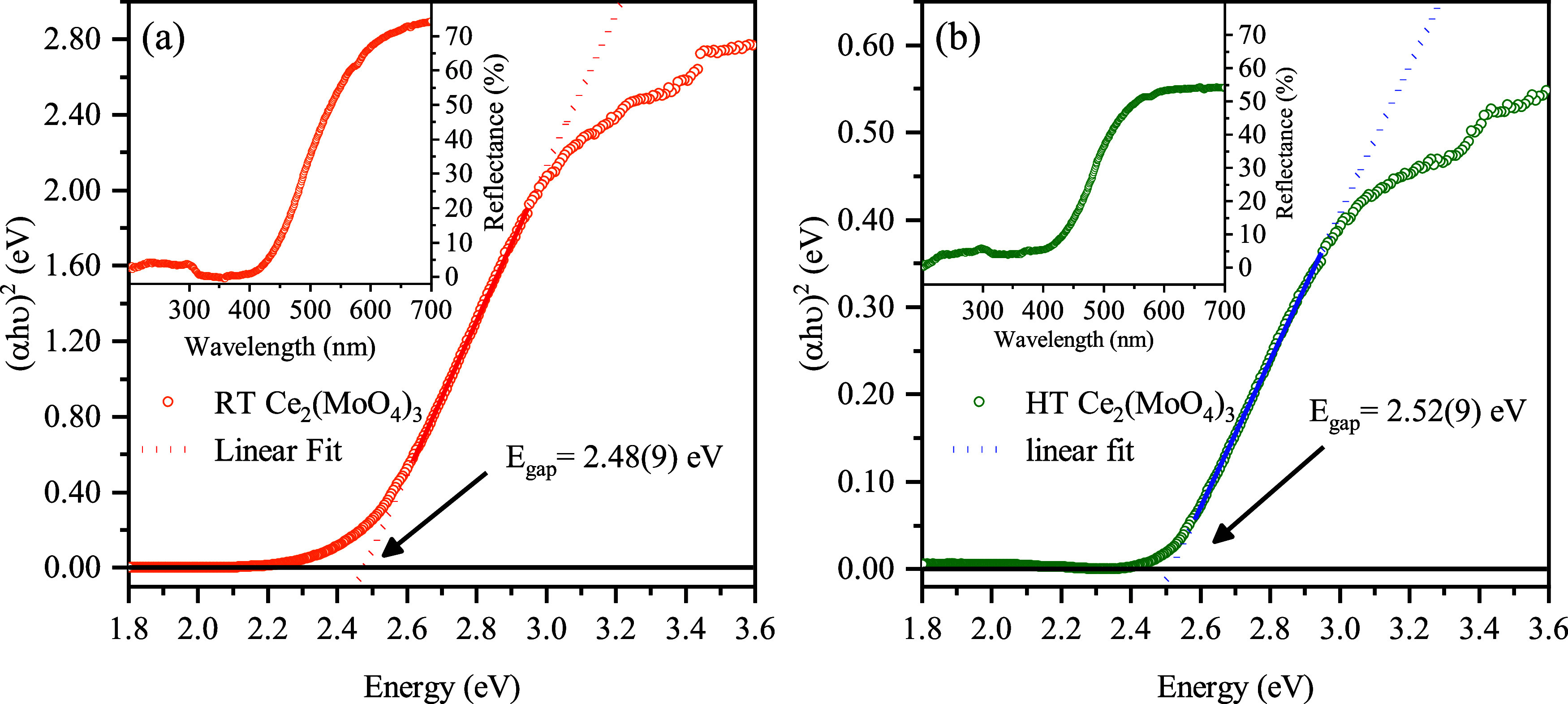
Estimated energy gap (*E*_gap_) for the Ce_2_(MoO_4_)_3_ sample (a) before and (b) after the heating process. Inset: UV–vis diffuse reflectance spectrum of the sample in the 200–700 nm range.

The reflectance data were converted into absorbance using the Kubelka–Munk function,^20^ and the optical band gap (*E*_gap_) was estimated using the method proposed by Wood and Tauc.^34,35^ The main plots shown in Figure 6a,b display the calculated *E*_gap_ value for the CMO sample, where we assumed direct transitions (*n* = 1/2) within the band structure.^29^ By extrapolating the linear region of the plot to (α*h*ν)^2^ = 0, energy gaps of 2.48(9) and 2.52(9) eV were obtained for the RT and HT materials, respectively. Those values align with the previous reports for CMO,^20,28,33,36^ suggesting its suitability for photocatalyst applications.

Recent work on rare-earth niobates carried out by Garg et al.^37^ revealed that the Kubelka–Munk–Tauc method employed here tends to underestimate the energy bang gap values by about 0.3 eV and, as such, the apparent increase from 2.48 (RT) to 2.52 (HT) falls within the uncertainty associated with the method itself. This suggests that the observed variation is not statistically significant and that the *E*_gap_ values remain effectively unchanged during the heating cycle.

The optical band gap energy of Ce_0.667_MoO_4_ (≈2.50 eV) is considerably smaller than typical values reported for scheelite-type molybdates, such as BaMoO_4_ (≈4.43 eV^38^), CdMoO_4_ (≈3.71 eV^38^), PbMoO_4_ (≈3.45 eV^38^), CaMoO_4_ (≈ 3.91 eV^39^), as well as scheelite-type tungstates, such as BaWO_4_ (≈5.01 eV^40^), CdWO_4_ (≈3.28 eV^41^), PbWO_4_ (≈3.24 eV^42^), and CaWO_4_ (≈5.92 eV^40^). In molybdates, the dominant optical transitions are O 2p → Mo 4d (valence band maximum → conduction band minimum), whereas in tungstates, the conduction band is instead dominated by W 5d states. Projected density of states (PDOS) calculations performed by Monteseguro et al.^38^ show that in PbMoO_4_, the Pb 6s orbital hybridizes with O 2p, creating additional states closer to the Fermi level, thereby lowering *E*_gap_. In our cerium molybdate Ce_0.667_MoO_4_ material, the even smaller *E*_gap_ may likewise emerge from a similar hybridization of either Ce narrower 4f levels (generating states closer to the Fermi level) or wider Ce 5d (slightly altering states close to the bottom of the conduction band) with O 2p, while the Mo 4d state remains the main contributor to the conduction band minimum. A definitive assignment of orbital participation, however, requires orbital-resolved PDOS calculations or core/valence X-ray photoelectron spectroscopy (XPS)/resonant photoemission experiments.

### 3.5 Temperature-Dependent PXRD Analysis

The PXRD patterns obtained under low- and high-temperature conditions are shown in Figure S1a–d (blue dots) and Figure 7a–d (red dots), respectively. In both thermal cycles, diffraction peaks remain strong, sharp, and well-defined, with low background intensity, and without the appearance of additional peaks. We conclude that the crystallinity of the CMO sample remains intact throughout the entire thermal cycle. In the zoomed-in versions, however, we notice subtle structural changes in the high-temperature patterns (Figure 7c,d) that are not seen in the low-temperature patterns (Figure S1c,d).

**Figure 7:**
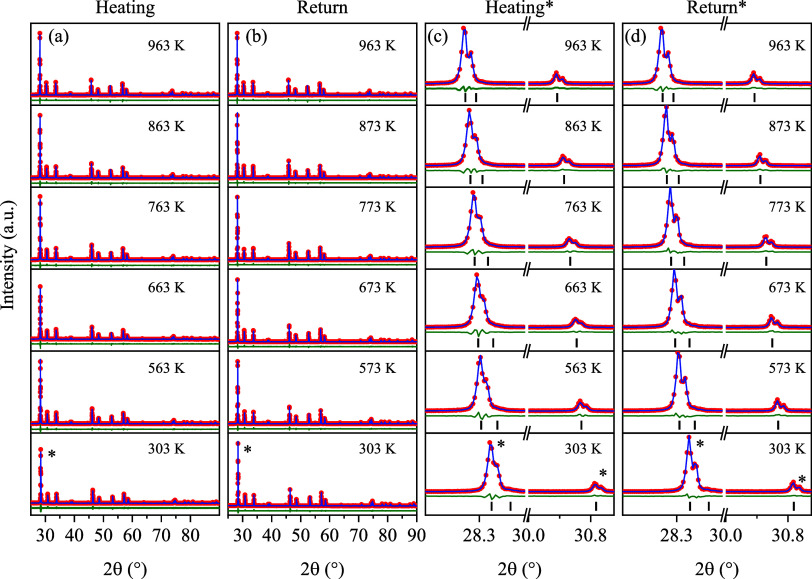
Powder X-ray diffraction patterns of the Ce_2_(MoO_4_)_3_ sample obtained during (a, c) heating and (b, d) upon return to room temperature at selected temperatures. Zoomed-in view of the 112, 103, and 004 Bragg peaks during (c) heating.

Namely, a slight broadening of the 112, 103, and 004 peaks can be observed at *T* > 673 K, suggesting the emergence of structural microstrain. Curiously, the broadening is seen to reverse upon return to room temperature (e.g., compare the 112 peak at 763 K in Figure 7c and at 773 K in Figure 7d). After the heating cycle, the positions of 112, 103, and 004 peaks have irreversibly shifted to slightly higher scattering angles (2θ°_return_ – 2θ°_heating_ = 0.0132(4), 0.0094(4), and 0.0060(4), respectively). These observations reveal that subtle, but permanent, structural changes underwent within the CMO sample during the heating cycle.

To quantify the observed structural behavior, the Rietveld refinement was performed on the diffraction patterns. All reflections were indexed (black bars) to the scheelite-type tetragonal structure in the *I*4_1_/*a* space group, corresponding to the Inorganic Crystal Structure Database (ICSD) card No. 423509.^9^ Figure 7a–d displays the calculated (blue lines) patterns and the *I*_obs_ – *I*_calc_ (green lines) plot. Examination of the temperature evolution of the refinement’s goodness-of-fit (χ^2^, Figure S2), reveals a pronounced dip in quality at *T* = 583 K coinciding with the onset of the observed deviations (peak broadening) in lattice behavior.

Figure 8a–c displays the temperature evolution of the lattice parameters in the 303–963 K range (see the Supporting Information, Tables S3 and S4, for numerical data). Between 303 and 583 K, the lattice parameters and unit cell volume exhibit a linear dependence with temperature, indicative of regular thermal expansion. However, beyond 583 K, a deviation from this linear trend was observed, persisting until 783 K (the region between vertical dashed lines), after which linearity was resumed up until 973 K. Upon return to room temperature, the lattice parameters maintain linear temperature dependence.

**Figure 8:**
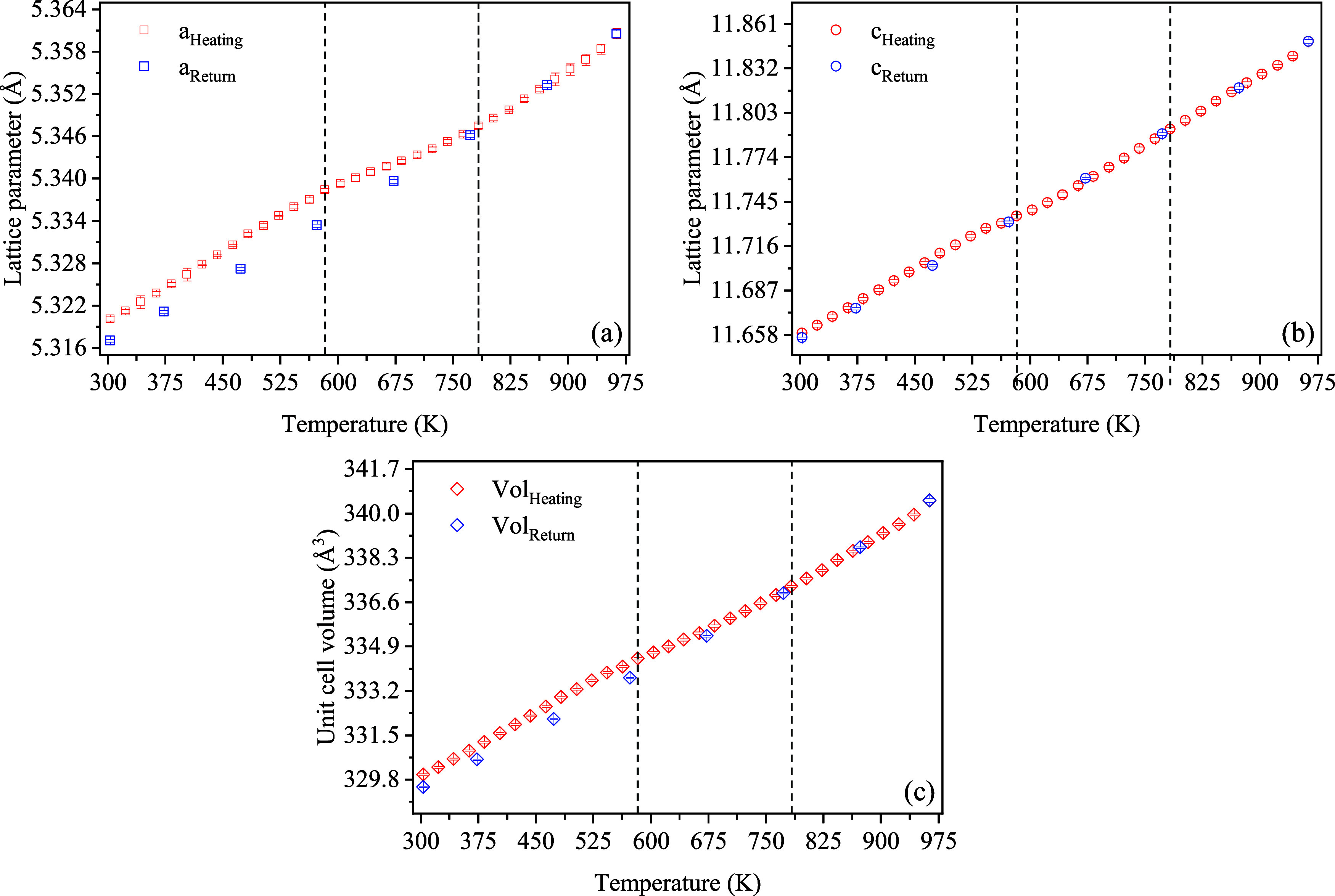
Lattice parameters *a* = *b* (a), *c* (b) and unit cell volume (c) of Ce_2_(MoO_4_)_3_ crystals vs temperature. The dashed line at *T* = 583 K marks a change in the initial trend, indicating structural modifications within the sample.

After the heating cycle, all lattice parameters are different from their corresponding values before the heat cycle (Δ*a* ≈ 0.0031 Å, Δ*c* ≈ 0.0030 Å; thus, ΔVol ≈ 0.4663 Å^3^ change in the volume), indicating structural hysteresis. This suggests the presence of a thermally induced structural anomaly in the sample, disrupting the linear expansion of the material. As such, the 583–783 K range may be interpreted as a transient regime, marking a structural adjustment toward a metastable configuration.

To obtain quantitative data regarding the sample’s thermal behavior, we perform dilatometry analysis. The relationship between the linear thermal expansion coefficients α*_ij_* of the room- and high-temperature phases and their respective strains *P_ij_* can be obtained by the second-rank symmetrical tensor:

2 $$P_{\mathrm{ij}}=\alpha_{\mathrm{ij}}\Delta T$$

where Δ*T* is the homogeneous change in temperature. Following the procedure described by Paufler et al.^43^ the tensor α*_ij_* has been constructed on the basis {*e*_1_, *e*_2_, *e*_3_}, where *c*||*e*_3_, b*||*e*_2_, and *e*_1_||*c* × *b*. The symmetry of the tetragonal system ensures the cancellation of off-diagonal terms, resulting in only diagonal components α*_ii_* being nonzero. The thermal expansion tensor was determined with linear least-squares refinement for three temperature intervals: α_ij_^1^, from 303 to 583 K, α_ij_^2^, from 783 to 963 K, and α_ij_^3^, from 963 to 303 K, upon return to room temperature. The intermediate region (583–783 K) was excluded from the linear thermal expansion analysis due to clear nonlinear behavior in the lattice parameters (Figure 8a–c), indicative of a structural anomaly or transition. The coefficients of volumetric expansion γ^1^, γ^2^, and γ^3^ are given by the traces of the α_ij_^1^, α_ij_^2^, and α_ij_^3^ tensors, respectively (see Supporting Information, Table S5). The linear fits for each temperature interval are displayed in Figure 9a–c.

**Figure 9:**
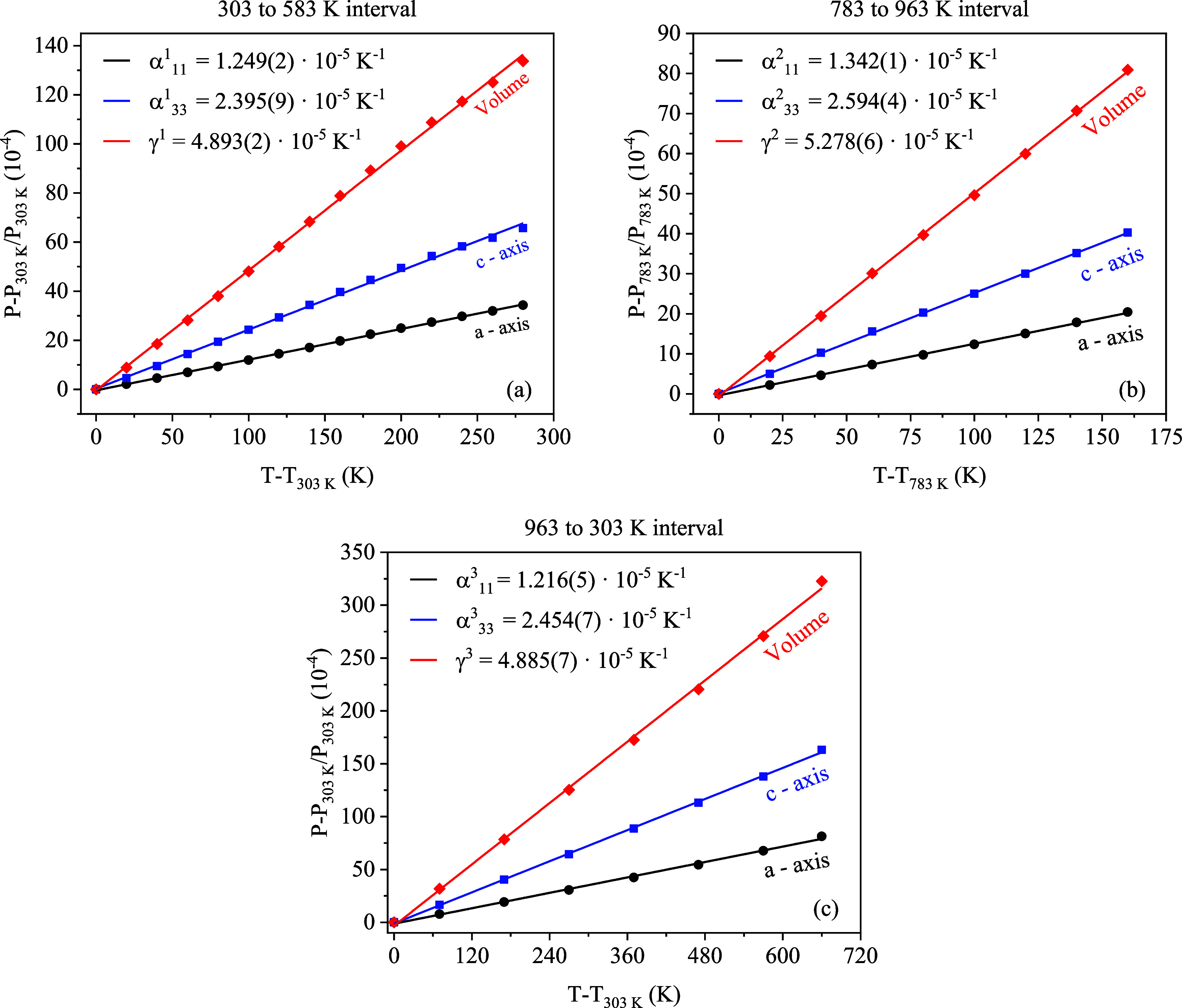
Linear fits of relative variations of lattice parameters *a* = *b*, *c*, and unit cell volume of the Ce_2_(MoO_4_)_3_ crystals as functions of temperature for the 303–583 K (a), 783–963 K (b) and 963–303 K (c) intervals.

The α_ij_^1^, α_ij_^2^, and α_ij_^3^ symbols represent the coefficients of linear expansion along the *ij*th axis of the unit cell for each temperature interval. The relative difference between the α_11_^k^, α_33_^k^, and the volumetric expansion coefficients γ*^k^* are displayed in Table 5.

**Table 5:** Relative Differences in the Thermal Expansion Coefficients of Ce_2_(MoO_4_)_3_ Crystals^a^

303–583 and 783–963 K temperature interval			303–583 and 963–303 K temperature interval		
*a* = *b*	*c*	Vol	*a* = *b*	*c*	Vol
$1-\frac{\alpha_{11}^{2}}{\alpha_{11}^{1}}$	$1-\frac{\alpha_{33}^{2}}{\alpha_{33}^{1}}$	$1-\frac{\gamma^{2}}{\gamma^{1}}$	$1-\frac{\alpha_{11}^{3}}{\alpha_{11}^{1}}$	$1-\frac{\alpha_{33}^{3}}{\alpha_{33}^{1}}$	$1-\frac{\gamma^{3}}{\gamma^{1}}$
–7.446%	–8.309%	–7.868%	2.722%	–2.463%	0.184%

a α_11_ for *a* = *b*, α_33_ for *c* directions and γ for volumetric thermal expansion, across the three temperature intervals, indicated by the superscripts: 1 (303–583 K), 2 (783–963 K), and 3 (963–303 K, cooling).

The coefficient of thermal expansion α*_ij_* quantifies the rate of expansion of the lattice along its *ij*th axis. The negative relative differences between α_ij_^1^, γ^1^, and α_ij_^2^, γ^2^, indicate pronounced increase in the expansion rate at high temperatures; however, upon return to room temperature, the positive relative difference between α_11_^1^, α_11_^3^ and γ^1^, γ^3^ indicate the reduction in the expansion rate of the a-parameter and the unit cell volume, respectively. Although the expansion rate of the *c*-parameter also decreased, it remains higher than its corresponding rate before 583 K.

The changes observed in the sample’s thermal response strongly suggest the emergence of a second structural phase of the CMO material at *T* ≈ 583 K, with distinct lattice parameters. This explains the deviation from the linear thermal behavior. At *T* ≈ 783 K, the thermal response resumes linear behavior, indicating that the system has reached a metastable equilibrium. Consequently, the diffraction patterns at *T* > 583 K contain contributions from both phases, and, therefore, the calculated α^2^, α^3^ and γ^2^, γ^3^ should be interpreted as apparent coefficients of thermal expansion, rather than the properties of a single homogeneous phase.

The temperature evolution of the polyhedron’s internal Mo–O and Ce–O bond lengths is displayed in Figure 10a, showing regular elongation with temperature. The ∠(O_3_–Ce–O_7_) and ∠(O_1_–Ce–O_4_) angles Figure 10b, however, exhibit opposite trends: the former narrows, while the latter widens. Interestingly, these angles become equal at *T* > 583 K, before diverging further. The temperature evolution of the other O–Ce–O and O–Mo–O angles, as well as the Ce–O–Mo polyhedral angles are displayed in Figures S3, S4, S5, and S6, respectively, remaining largely unchanged with temperature, apart from regular thermal expansion and consequently widening/shortening of some of the internal O–Ce–O angles. These observations suggest lack of pronounced local structural distortions that might be responsible for the anomalous behavior in the lattice parameter’s temperature evolution.

**Figure 10:**
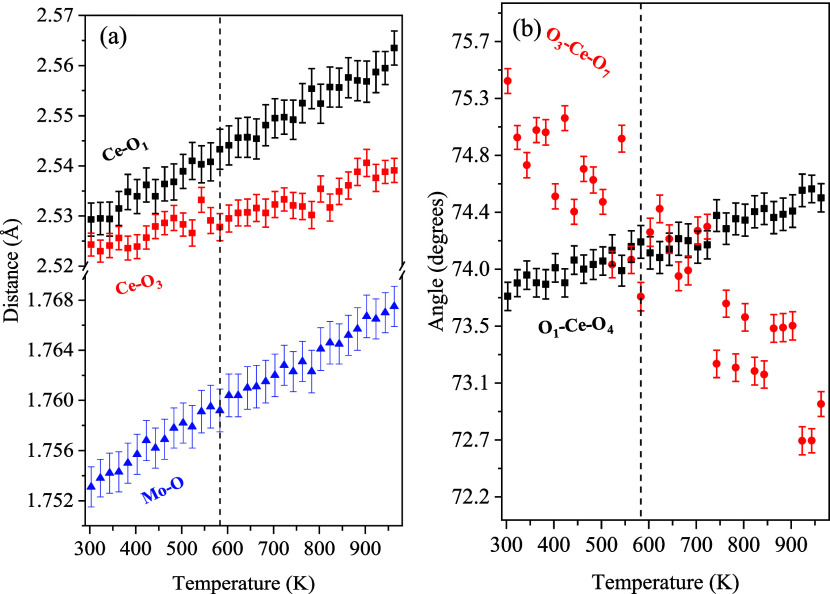
Temperature dependence of the internal polyhedral geometry in Ce_2_(MoO_4_)_3_. (a) Selected bond lengths, including Mo–O (blue triangles) and two distinct Ce–O bonds (black and red squares). (b) Selected O–Ce–O bond angles (black and red squares).

Consequently, we investigated the possibility that the anomaly may instead be associated with long-range effects, manifested as diffraction peak broadening. The sample-related contribution of to the peak’s broadness β*_hkl_* was obtained by removing the instrumental contribution β_ins_ from the observed broadening β_obs_ using the equation β*_hkl_* = (β_obs_^2^ – β_ins_^2^)^1/2^. The β_obs_ was obtained from the Rietveld refinement of the *hkl* reflections. Assuming that the strain and size contributions to peak broadening are independent of one another, we can calculate them using the Williamson–Hall^44^ (W–M) equation:

3 $$\cos\left(\theta_{\mathrm{hkl}}\right)\beta_{\mathrm{hkl}}=K\lambda /D+4\varepsilon \;\sin\left(\theta_{\mathrm{hkl}}\right)$$

where *K* is the Scherrer shape factor (*K* = 0.9),^45^ λ is the wavelength of the Cu Kα_1_ radiation, *D* is the crystallite size, and ε is the sample’s microstrain. Plotting the LHS of the eq 1 as a function of 4 sin θ*_hkl_*, we obtained the W–M plot, from which the isotropic microstrain and crystallite size parameters can be respectively estimated with the slope and the intercept of the linear fit to the plot.^46^ The W–M analysis was carried out using the 004 peak, and the temperature evolution of the sample’s microstrain and crystallite size is displayed in Figure 11a and 11b, respectively.

**Figure 11:**
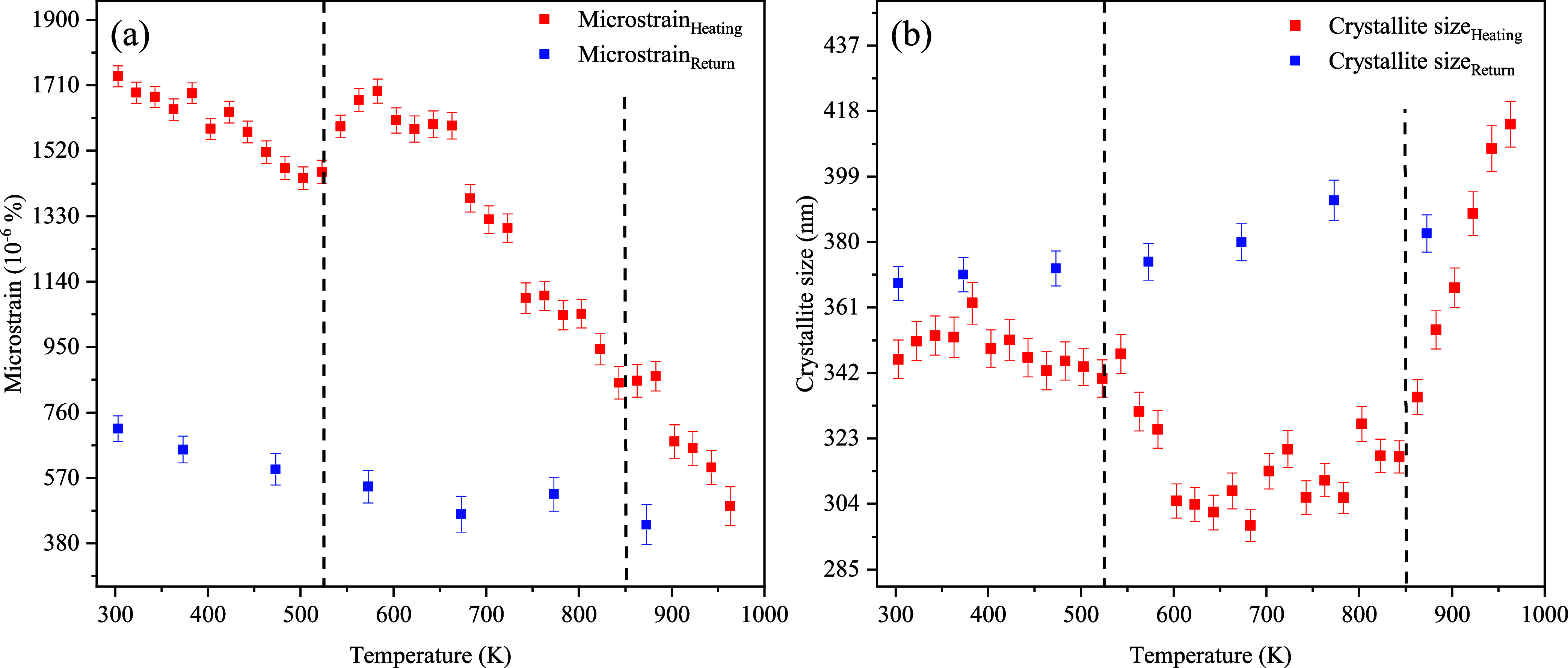
Evolution of the microstrain (a) and crystallite size (b) in Ce_2_(MoO_4_)_3_ across the full temperature range. The dashed lines indicate the points at which changes in the tendency occur, marking shifts in the sample’s structural stabilization.

As the temperature increases, the sample’s microstrain initially decreases, as is typically expected. However, at *T* ≈ 543 K, a distinct rise is observed, after which the decreasing trend resumes. Upon returning to room temperature, the microstrain gradually increases again, although it remains significantly lower than the values observed during the heating cycle, indicating a relaxation of the strains/defects present in the sample’s original state. The anomalous microstrain behavior at *T* > 543 K closely matches the temperature beyond which we see the deviation from linear behavior in the lattice parameters.

A similar trend is observed in the sample’s crystallite size (Figure 11b). It remains approximately constant up to *T* ≈ 563 K, then it steadily decreases until *T* ≈ 543 K, after which it grows to a maximum at 963 K. Upon return to room temperature, the crystallite size plateaus at values slightly above the preheating baseline, mirroring the hysteresis seen in the microstrain. Emergence of clear anomalous trends in the samples crystallite size and microstrain and overall stability of the internal bond angles and lengths point to long-range strain relaxation mechanisms intrinsic to the bulk sample, rather than localized effects.

The anomalous behavior of microstrain and crystallite size between 523 and 843 K closely correlates with the nonlinear thermal response in lattice parameters and thermal expansion coefficients between 583 and 743 K during heating. Saha et al.^12^ reported this exact behavior in the scheelite-type lithium–cerium molybdate LiCe­(MoO_4_)_3_ (LCM) using high-resolution synchrotron X-ray diffraction. An isostructural phase transition was observed above 673 K, evidenced by the appearance of low-intensity diffraction peaks near the pre-existing ones and an increase in peak broadening in the temperature range from 323 to 673 K temperature range. By analogy, the parallel increase in peak broadening and microstrain in our Ce_2_(MoO_4_)_3_ sample strongly suggests the same phase-nucleation-driven stress: that is, as nuclei of the new phase form, they locally distort the lattice, producing inhomogeneous strain that broadens diffraction peaks. Its partial irreversibility on the return to room temperature, as evidenced by the persistent peak position shifts, changes in the thermal expansion tensors, as well as lower microstrain values after the heating cycle, points toward the stabilization of a metastable isostructural phase (tetragonal *I*4_1_/*a*, with modified cell dimensions).

The absence of Bragg reflections belonging to the new phase aligns with the thermodynamic model by Christy et al.^4^ The existence of multiple local minima in the free-energy surface around transition points allows the coexistence of structurally similar states, and the discontinuities in the observed states are blurred by limited instrumental resolution. Indeed, the additional reflections observed by Saha and coauthors in their study^12^ were only visible with synchrotron XRD, and not in their home-laboratory data. Thus, the lack of new reflections in our data does not rule out minor phase segregation.

### 3.6 Temperature-Dependent Raman Spectroscopy Analysis

To gather further evidence of the isostructural phase transition, Raman spectra of the CMO sample were measured in the 298–998 K temperature range. Several key features associated with temperature-induced changes in the CMO sample are observed in Figure 12a. As the temperature rises up, the Raman-active modes shift slightly toward lower wavenumbers (and, therefore, higher wavelengths), reflecting the impact of thermal motion on phonon vibrations. Increased peak broadening can be observed in the 150–300 cm^–1^ region (associated with the lattice and Ce^3+^ translational modes) above 623 K, which may be attributed to an increased disorder or strain due to the onset of structural changes or enhanced anharmonic phonon interactions. Furthermore, we observe the abrupt emergence of a Raman band in the symmetric and antisymmetric bending regions around 452 cm^–1^ at 848 K, indicated by the arrow in Figure 12a. With further heating, the intensity of this band increases significantly, until it becomes comparable to the 318 cm^–1^ mode (peak 11) at 998 K, while also shifting progressively to lower wavenumbers. When the sample is cooled back to room temperature, the band persists with reduced intensity, shifting back upward toward higher wavenumbers, pointing to irreversible changes in the sample.

**Figure 12:**
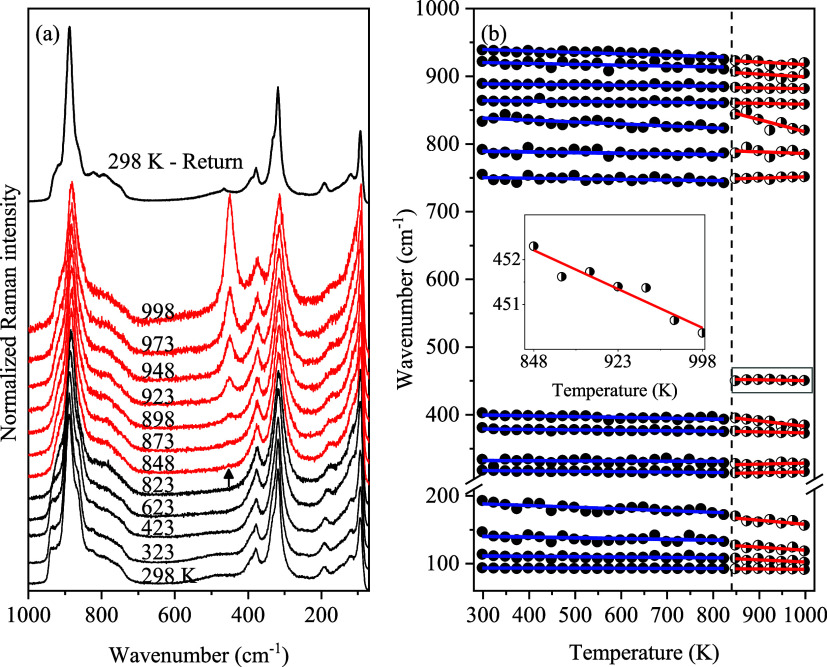
(a) Raman spectra of the Ce_2_(MoO_4_)_3_ sample measured over the 293–993 K temperature range, in the 1000–150 cm^–1^ spectral range. The arrow marks the appearance of the 452 cm^–1^ mode at 848 K. The spectrum at 298 K (after heating) at the top highlights the irreversibility of the isostructural phase transition. (b) Wavenumber vs temperature plot for the stretching, bending, and lattice modes. Solid blue and red lines represent linear fits for the room-temperature and high-temperature phases, respectively. The vertical dashed line marks the temperature where the additional mode is observed. Inset displays evolution of the new mode with temperature.

To obtain quantifiable information on the temperature evolution of the observed bands, the Raman spectra were fitted using a Voigt convolution function within the Fityk software.^47^ Figure 12b displays the wavenumbers of the observed bands as a function of the temperature. The frequency of the additional band coincides with the frequency of the F_2g_ mode of CeO_2_ (ω ≈ 464 cm^–1^)^48^, and it could easily be misinterpreted as evidence of a partial chemical decomposition at high temperatures. However, we note that (i) the high-temperature PXRD patterns in Figure 7a–d show no evidence of Bragg peaks from any crystalline decomposition products associated with cerium oxide (CeO_2_) phases; moreover, (ii) the reduced intensity of the additional mode in the room-temperature spectrum obtained after the heating cycle is inconsistent with how partial chemical decomposition works.

Additionally, rare-earth elements are prone to photoluminescence (PL) emission occurring in Raman-active zones due to their 4f electronic configuration. This raises the possibility that the additional band observed in Figure 12a might not be a Raman-active mode. Due to being strongly dependent on the excitation source, a PL originated band should disappear when the excitation wavelength is changed.^49^ To examine this, we measured the Raman spectrum of CMO powder using a red laser (λ = 633 nm) in addition to the green laser (λ = 532 nm) employed in our previous measurements. Figure 13a,b presents the spectra collected after and before the heating treatment, respectively. With both laser lines, the additional band persists at ∼452 cm^–1^, as shown in the insets, independently of the excitation wavelength. This observation rules out PL as the origin of the band.

**Figure 13:**
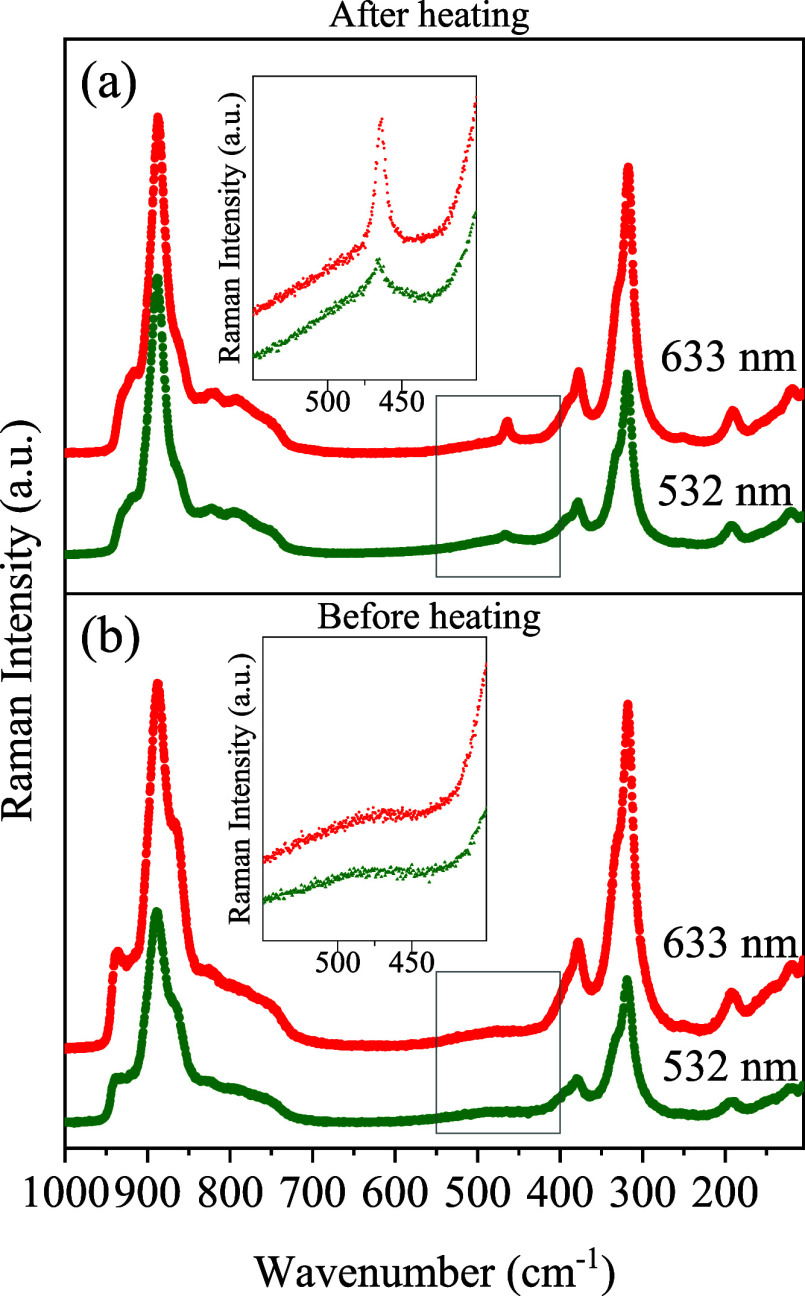
Raman spectra of the Ce_2_(MoO_4_)_3_ sample acquired with 532 nm (green circles) and 633 nm (red circles) excitation lasers. Spectra were collected (a) after heating and (b) before heating. Inset graphs are zoomed-in versions of main plots, highlighting the persistence of the 452 cm^–1^ mode after heating treatment.

Emergence of this additional band was previously reported by Saha et al.^12^ and Moura et al.^13^ for the tetragonal scheelite-type LiCe­(MoO_4_)_2_ (LCM) and NaCe­(MoO_4_)_2_ (NCM) materials, respectively. In their high-temperature Raman scattering studies, the 452 cm^–1^ band was observed at 748 and 763 K, with wavenumbers ∼440 and ∼458 cm^–1^ for the LCM and NCM materials, respectively, where it was attributed to a Raman-active vibrational mode. In the spectra obtained for our CMO material, the same band emerges above 848 K, exhibiting phonon-like temperature dependence: shifting toward lower wavenumber at heating and higher wavenumbers at room temperature (Figure 12a). Taken together, these observations demonstrate that the band is likewise an intrinsic vibrational mode of CMO, rather than an extrinsic artifact. Furthermore, its appearance only at high temperature indicates that it becomes Raman-active as a consequence of a structural change in the lattice.

The convergence of evidence from the PXRD patterns (the lattice parameter, microstrain, and crystallite size anomalies, similar to the report by Saha et al.^12^) and the Raman spectra (emergence of the vibrational mode and peak broadening in the lattice-attributed vibrational modes, similar to report by Moura et al.^13^) confirms the occurrence of a displacive, weakly first-order isostructural phase transition in our CMO sample. Under this consideration, the intensity increase with temperature of the 452 cm^–1^ mode aligns with that of the study by Saha et al.^12^ proposed the mechanism of phase nucleation, consequently explaining the anomalies observed in the sample’s microstrain and crystallite size. It should be noted that the emergence of this vibrational mode is not a universal feature among scheelite-type molybdates. A recent report by Ramarao et al.^14^ showed a similar phase transition in calcium molybdate CaMoO_4_, another tetragonal scheelite-structured material. No new vibrational modes were observed during the phase transition, as revealed by temperature-dependent Raman spectroscopy. This suggests that the appearance of this mode during the transition may be a distinctive characteristic of cerium-based scheelite-type molybdates.

The wavenumbers ω as functions of temperature, *T*, were fitted to a linear phonon decay model^50^ as described by eq 4.

4 $$\omega \left(T\right)=\omega_{0}+\eta T$$

The wavenumber intercept ω_0_, along with the first-order $\left(\frac{\partial \omega }{\partial T}\right)$ temperature coefficient η, obtained from the fitting process, are listed in Table 6.

**Table 6:** Observed Wavenumber ω_obs_ and Estimation of Wavenumber Intersect ω_0_, and Temperature Coefficient η of Vibrational Modes of Ce_2_(MoO_4_)_3_ Crystals across the Temperature Intervals 298–823 and 848–998 K

	Room-temperature phase (298–823 K)			High-temperature phase (848–998 K)		
Peak no.	ω_obs_ at 298 K (cm)	ω_0_ (cm^–1^)	η (cm^–1^/K)	ω_obs_ at 848 K (cm^–1^)	ω_0_ (cm^–1^)	η (cm^–1^/K)
1	937	946	–0.021	925	955	–0.037
2	920	924	–0.013	903	939	–0.040
3	889	891	–0.007	884	892	–0.011
4	864	866	–0.006	865	872	–0.013
5	839	847	–0.029	836	999	–0.181
6	789	792	–0.010	801	811	–0.025
7	749	753	–0.009	763	733	0.019
new				452	462	–0.012
8	401	403	–0.012	395	461	–0.078
9	381	381	–0.006	376	389	–0.016
10	331	333	–0.002	328	316	0.013
11	317	319	–0.005	314	305	0.011
12	190	196	–0.025	170	219	–0.061
13	143	144	–0.011	133	164	–0.044
14	112	113	–0.005	108	135	–0.032
15	93	94	–0.001	92	98	–0.007

All η coefficients for the room-temperature phase have negative values, indicating a consistent trend of phonon softening induced by a high temperature. In the high-temperature phase, most of the observed η coefficients decrease in magnitude, with the exception of three vibrational modes (peaks 7, 10, and 11), which exhibit positive coefficients. Such changes are consistent with the expected impact of structural phase transitions on vibrational modes, where changes in phonons indicate shifts in the material properties.

Our PXRD analysis indicates that the phase transition settles at 783 K, while our Raman spectroscopy analysis indicates that the low-temperature phase persists until about 848 K. This 65 K difference is similarly seen in the report by Saha et al.:^12^ the additional mode was observed at 490 °C, while the additional reflections of the high-temperature isostructural phase were observed at 400 °C, for a 90 K difference. Such discrepancy arises from the intrinsic characteristics of each technique. The PXRD patterns are resultant of probing over a large portion of the sample, averaging structural information over many grains and domains, and is therefore sensitive to long-range structural changes; nevertheless, subtle trend changes such as peak shifting or broadening may still provide information on temperature-induced structural variations. In contrast, Raman spectroscopy examines a highly localized area, limited by the laser spot size, typically a few micrometers, often focusing on larger grains to maximize signal quality. Such localized probing can capture regions where the phase transition occurs more gradually or that exhibit greater thermal stability than the bulk average detected by PXRD. Consequently, the transition temperature observed by Raman spectroscopy may appear higher than that determined from PXRD measurements.

## 4 Conclusions

This study explored the high-temperature behavior of the scheelite-type cerium molybdate Ce_2_(MoO_4_)_3_, synthesized via hydrothermal synthesis. Characterization was carried out using powder X-ray diffraction, Raman spectroscopy, and UV–vis diffuse reflectance. The crystalline nature of the sample was confirmed, with no evidence of secondary phases or impurities. The sample crystallized in the *I*4_1_/*a* space group, in agreement with the previously reported structures, thereby confirming the success of the synthesis. SEM micrographs revealed irregular size and morphology with no amorphous aggregates. Temperature-dependent XRD patterns displayed no new diffraction peaks, indicating unchanged crystal symmetry. However, several anomalies were detected at 583 K, including shifts in lattice parameter trends, an increase in the microstrain, and a decrease in the crystallite size, all indicative of structural changes consistent with a weakly first-order isostructural phase transition, as previously reported.^12,13^ Temperature-dependent Raman spectroscopy further corroborated these findings, revealing broadening of lattice-mode-related peaks and the emergence of a new 452 cm^–1^ vibrational mode at 848 K. Persistence of structural (lattice parameters smaller after heating) and vibrational changes (452 cm^–1^ mode) confirms the irreversible nature of this phase transition. These results highlight the interplay between the structural and vibrational properties of materials undergoing IPTs. The observed anomalies in PXRD and the emergence of new Raman modes underscore the sensitivity of local and long-range probes to subtle structural changes. Understanding such transitions is crucial, as they can influence electronic, optical, and mechanical properties, offering guidance for future studies aiming to tailor these materials for specific functional applications.

## Supplementary Material



## Notes

Bond lengths and angles calculated from PXRD data, low-temperature PXRD characterization, and thermal expansion data (PDF)
