# Supplementary material for: High-Temperature Isostructural Phase Transition in Ce2(MoO4)3: A Rare Phenomenon Investigated through X‑ray Diffraction and Raman Scattering
Source: ACS Omega. 2026 Jan 6;11(2):2455–68. doi: 10.1021/acsomega.5c03153 (PMC12824781; doi:10.1021/acsomega.5c03153)
Supplement: Supplementary file 1 [file ao5c03153_si_001.pdf]

Supplementary information

High-temperature Isostructural Phase Transition in $Ce_2(MoO_4)_3$: A Rare Phenomenon Investigated Through X-ray Diffraction and Raman Scattering

Zeyna dos Santos Viegas[a], Alan Silva de Menezes[a], Cleânio Luz-Lima[b], Paulo de Tarso Cavalcante Freire[c], Clenilton Costa Santos[a], and João Victor Barbosa Moura[a]*

[a] *Departamento de Física, Centro de Ciências Exatas e Tecnologia, Universidade Federal do Maranhão, CEP 65080-800, São Luís, MA, Brazil*

[b] *Departamento de Física, Campus Ministro Petrônio Portella, Universidade Federal do Piauí, CEP 64049-550, Teresina, PI, Brazil*

[c] *Departamento de Física, Campus do Pici, Universidade Federal do Ceará, CEP 60455-760, Fortaleza, CE, Brazil*

*Email: jvb.moura@ufma.br

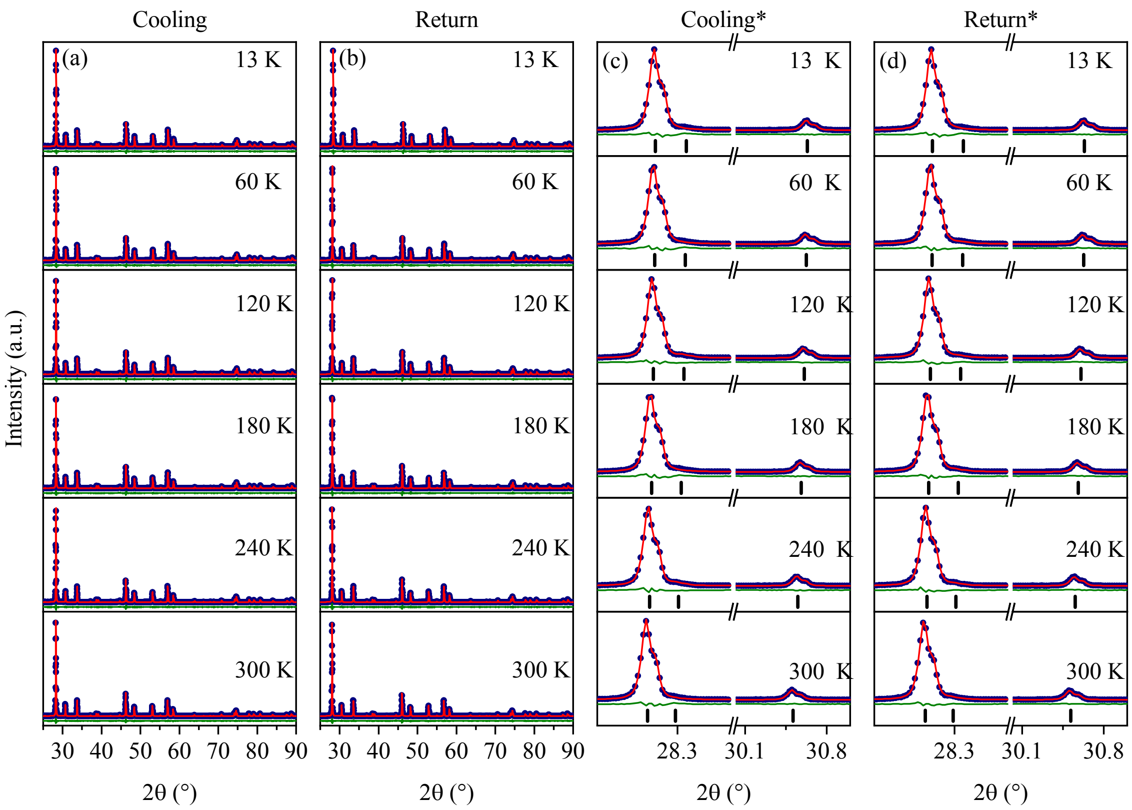

Figure S1: Powder X-ray diffraction patterns of the $Ce_2(MoO_4)_3$ (ICSD 423509) sample obtained during ((a) & (c)) cooling and ((b) & (d)) upon return to room temperature at selected temperatures. Zoomed-in view of the 112, 103, and 004 Bragg peaks during (c) cooling and (d) upon return to room temperature. Minimal peak shifts are seen.

Table S1: Bond angles between the cerium and oxygen atoms for the RT $Ce_2(MoO)_4$ material

Ce-O bond angles (degrees)	∢(O4-Ce-O8) = ∢(O1-Ce-O5) = ∢(O2-Ce-O7) = ∢(O3-Ce-O6)	147.66(13)
	∢(O2-Ce-O6) = ∢(O1-Ce-O8)	138.10(12)
	∢(O3-Ce-O4) = ∢(O4-Ce-O7) = ∢(O5-Ce-O7) = ∢(O3-Ce-O5)	129.5(2)
	∢(O1-Ce-O2) = ∢(O1-Ce-O6) = ∢(O2-Ce-O8) = ∢(O6-Ce-O8)	97.35(13)
	∢(O2-Ce-O4) = ∢(O5-Ce-O6) = ∢(O1-Ce-O7) = ∢(O3-Ce-O8)	77.2(2)
	∢(O3-Ce-O7) = ∢(O4-Ce-O5)	74.19(11)
	∢(O1-Ce-O4) = ∢(O5-Ce-O8) = ∢(O2-Ce-O3) = ∢(O6-Ce-O7)	74.07(12)
	∢(O2-Ce-O5) = ∢(O4-Ce-O6) = ∢(O1-Ce-O3) = ∢(O7-Ce-O8)	69.63(14)

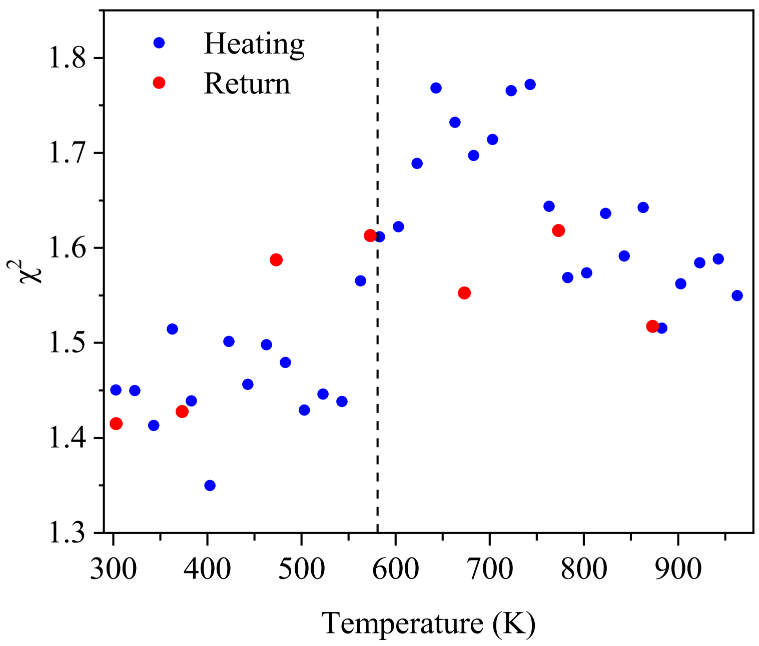

Figure S2: Temperature evolution of the χ^2 of the refined diffraction patterns of the $Ce_2(MoO_4)_3$ sammple during heating (blue dots) and upon return to room temperature (blue dots). The dashed line at T = 583 K marks the point beyond which the quality of the refinement decreases.

Table S2: Relative deviation of $Ce_2(MoO_4)_3$ material's internal bond angles from $CaWO_4$ scheelite [19]). The notation d(O_1 – A) stands for distance between O_1 and atom A (site 4b) or B (site 4a), while the notation ∢ stands for angle. Atom numbering correspond to Figure 2.

	A = Ce<br>B = Mo	A = Ca [19]<br>B = W [19]	Δ (%)
∢(O4-A-O8)	149.8(2)	152.25(7)	-3.1
∢(O2-A-O6)	136.5(2)	134.28(6)	2.8
∢(O3-A-O4)	127.7(2)	126.1(1)	2.6
∢(O1-A-O2)	97.9(2)	98.6(8)	-1.4
∢(O2-A-O4)	77.4(2)	79.7(3)	-3.3
∢(O3-A-O7)	77.05(15)	76.6(3)	-3.3
∢(O1-A-O4)	73.5(2)	73.2(2)	1.1
∢(O2-A-O5)	68.75(14)	68.5(8)	1.5

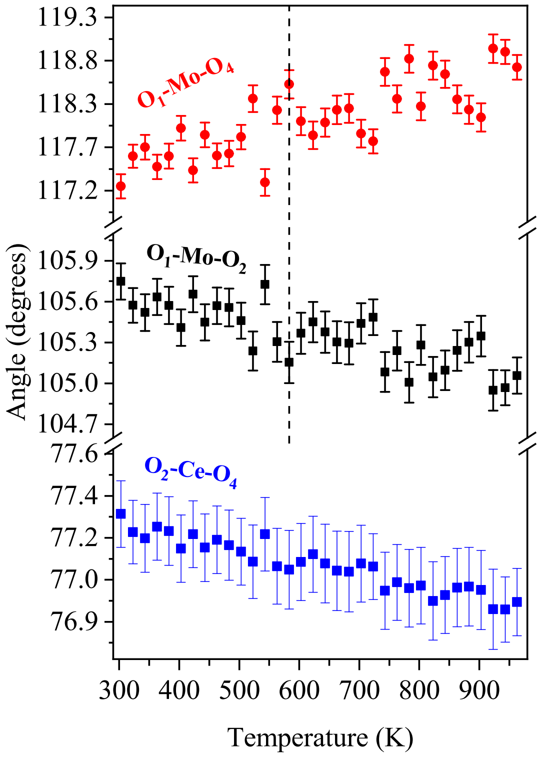

Figure S3: Temperature evolution of the internal O-Mo-O angles, as well as the ∢(O_2-Ce-O_4) angle of the tetrahedral and dodecahedral units in the $Ce_2(MoO_4)_3$ material.

Table S3: Lattice parameters of $Ce_2(MoO_4)_3$ crystals during heating.

Heating			
Temp. (K)	a = b (Å)	c (Å)	Vol. (Å^3)
303	5.320(3)	11.659(7)	329.991(3)
323	5.321(3)	11.664(2)	330.271(3)
343	5.322(9)	11.670(2)	330.599(3)
363	5.323(3)	11.676(8)	330.911(3)
383	5.325(3)	11.682(9)	331.239(3)
403	5.326(9)	11.687(3)	331.578(3)
423	5.327(1)	11.693(7)	331.918(3)
443	5.329(1)	11.699(5)	332.249(3)
463	5.330(1)	11.705(2)	332.598(3)
483	5.332(3)	11.711(3)	332.965(3)
503	5.333(3)	11.717(1)	333.274(3)
523	5.334(1)	11.722(7)	333.612(3)
543	5.336(3)	11.727(5)	333.911(3)
563	5.337(3)	11.731(6)	334.136(4)
583	5.338(3)	11.736(4)	334.443(4)
603	5.339(4)	11.740(4)	334.675(4)
623	5.340(4)	11.744(5)	334.905(4)
643	5.340(4)	11.749(1)	335.163(4)

663	5.341(4)	11.755(5)	335.425(4)
683	5.342(3)	11.761(5)	335.694(4)
703	5.343(3)	11.767(9)	335.982(3)
723	5.344(3)	11.773(7)	336.254(3)
743	5.345(3)	11.780(1)	336.566(3)
763	5.346(3)	11.786(4)	336.871(3)
783	5.347(3)	11.792(6)	337.206(3)
803	5.348(3)	11.798(8)	337.504(3)
823	5.349(1)	11.804(7)	337.828(3)
843	5.351(3)	11.811(7)	338.210(3)
863	5.352(3)	11.817(9)	338.560(3)
883	5.354(9)	11.822(2)	338.908(3)
903	5.355(8)	11.828(2)	339.253(3)
923	5.356(8)	11.834(5)	339.586(3)
943	5.358(7)	11.840(8)	339.952(3)
963	5.360(7)	11.849(7)	340.507(3)

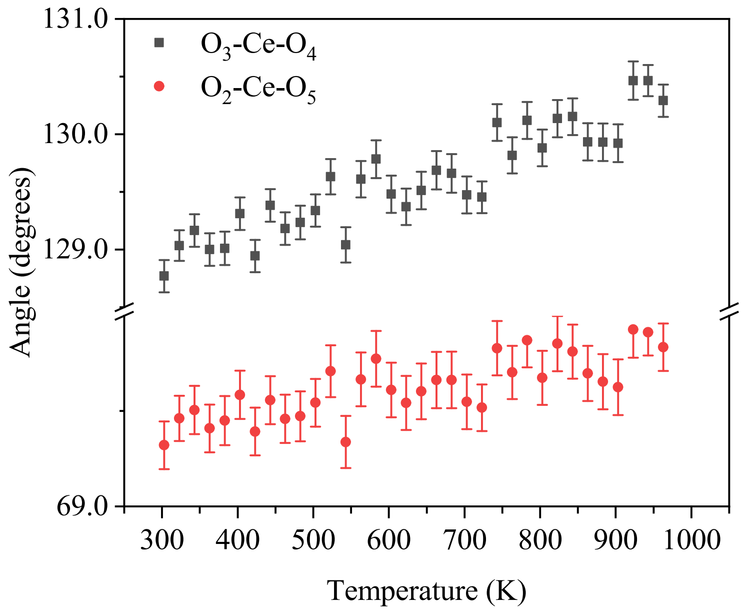

Figure S4: Temperature evolution of the internal ∢(O_3-Ce-O_4) and ∢(O_2-Ce-O_5) angles of the dodecahedral units in the $Ce_2(MoO_4)_3$ material.

Table S4: Lattice parameters of $Ce_2(MoO_4)_3$ crystals during return to room temperature.

Return to room temperature			
Temp. (K)	a = b (Å)	c (Å)	Vol. ($Å^3$)
303	5.317(2)	11.656(6)	329.525(2)
373	5.321(3)	11.675(9)	330.575(6)
473	5.327(3)	11.703(1)	332.128(9)
573	5.333(4)	11.731(5)	333.703(4)

673	5.339(4)	11.760(5)	335.306(3)
773	5.346(4)	11.789(7)	336.956(1)
873	5.353(6)	11.819(2)	338.703(7)
963	5.360(7)	11.849(7)	340.507(3)

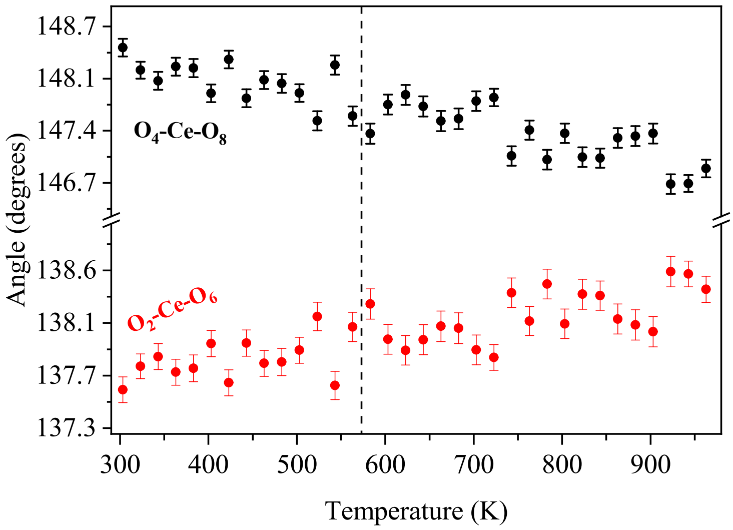

Figure S5: Temperature evolution of the internal ∢(O_2-Ce-O_6) and ∢(O_4-Ce-O_8) angles of the dodecahedral units in the $Ce_2(MoO_4)_3$ material.

Table S5: Thermal expansion tensors constructed based on the linear fits (Figure 7). Superscripts correspond to temperature intervals of fit: (1) 303–583 K, (2) 783–963 K, and (3) 963–303 K (return to room temperature).

Thermal expansion tensors	Volumetric expansion coefficients
$\alpha_{ij}^{1} = \begin{pmatrix} 1.249(2) & 0 & 0 \\ 0 & 1.249(2) & 0 \\ 0 & 0 & 2.395(9) \end{pmatrix} \times 10^{-5}\ K^{-1}$	$\gamma^{1} = 4.893(2) \times 10^{-5}\ K^{-1}$
$\alpha_{ij}^{2} = \begin{pmatrix} 1.342(1) & 0 & 0 \\ 0 & 1.342(1) & 0 \\ 0 & 0 & 2.594(4) \end{pmatrix} \times 10^{-5}\ K^{-1}$	$\gamma^{2} = 5.278(6) \times 10^{-5}\ K^{-1}$
$\alpha_{ij}^{3} = \begin{pmatrix} 1.215(5) & 0 & 0 \\ 0 & 1.215(5) & 0 \\ 0 & 0 & 2.454(7) \end{pmatrix} \times 10^{-5}\ K^{-1}$	$\gamma^{3} = 4.884(7) \times 10^{-5}\ K^{-1}$

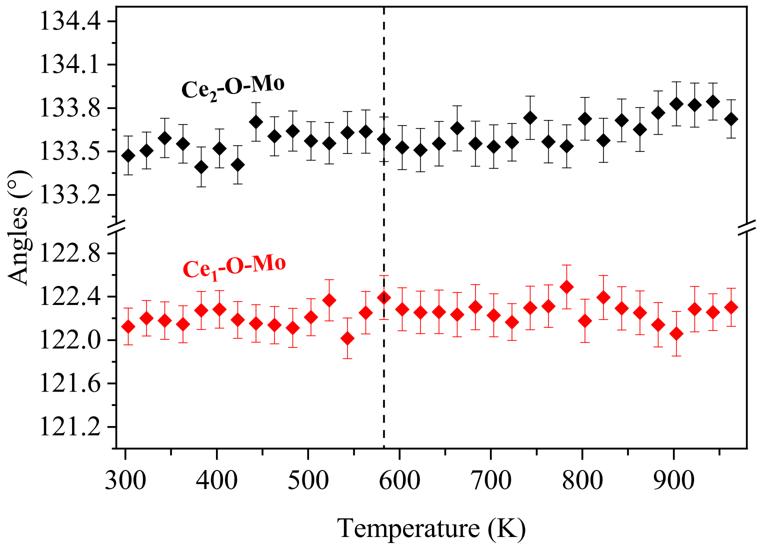

Figure S6: Temperature evolution of the external Ce-O-Mo angles between the dodecahedral and tetrahedral units in the $Ce_2(MoO_4)_3$ material.
